# MLH1 Deficiency Induces Cetuximab Resistance in Colon Cancer via Her‐2/PI3K/AKT Signaling

**DOI:** 10.1002/advs.202000112

**Published:** 2020-05-26

**Authors:** Ying Han, Yinghui Peng, Yaojie Fu, Changjing Cai, Cao Guo, Shanshan Liu, Yiyi Li, Yihong Chen, Edward Shen, Kexin Long, Xinwen Wang, Jian Yu, Hong Shen, Shan Zeng

**Affiliations:** ^1^ Department of Oncology Xiangya Hospital Central South University Changsha Hunan 410008 China; ^2^ Department of Pathology University of Pittsburgh School of Medicine Pittsburgh PA 15213 USA; ^3^ Key Laboratory for Molecular Radiation Oncology of Hunan Province Xiangya Hospital Central South University Changsha Hunan 410008 China; ^4^ Department of Life Science McMaster University Hamilton ON L8S 4L8 Canada; ^5^ National Clinical Research Center for Geriatric Disorders Xiangya Hospital Central South University Changsha Hunan 410008 China

**Keywords:** cetuximab, colon cancer, HER‐2, mutL homolog 1, phosphoinositide 3‐kinases pathway

## Abstract

The rapid onset of resistance to cetuximab (CTX) limits its clinical utility in colorectal cancer (CRC) patients. This study aims to understand a potential role of mismatch repair gene mutL homolog 1 (MLH1) in CTX response. Functional analysis of MLH1 in Her‐2/phosphoinositide 3‐kinases (PI3K)/PKB protein kinase (AKT)‐regulated CTX sensitivity is performed using human CRC specimens, CRC cell lines with different MLH1 expression levels, and a subcutaneous xenograft model. Overexpression, knockdown, small interfering RNA, and inhibitors are used to examine the role of MLH1 and HER‐2 downstream signaling and apoptotic targets in CTX sensitivity. Reduced MLH1 expression is correlated with unfavorable prognosis in cetuximab‐treated patients. *MLH1* loss decreases CTX sensitivity through Her‐2/PI3K/AKT signaling and apoptosis resistance in culture and in xenografts, while *MLH1* overexpression increases CTX sensitivity. Blocking Her‐2 signaling increases CTX sensitivity of microsatellite instability CRC in vitro and in vivo. MLH1 loss induces activation of Her‐2/PI3K/AKT signaling and leads to cetuximab resistance in colon cancer.

## Graphical abstract

Mismatch repair (MMR) genes deficiency can drive genomic instability and therapeutic resistance in colorectal cancer (CRC). Nevertheless, many of the mechanisms of MMR affecting drug resistance in CRC are still enigmatic. It is revealed that mutL homolog 1 deficiency decreases cetuximab sensitivity through Her‐2/phosphoinositide 3‐kinases/PKB protein kinase signaling, which provides a mechanism of overcoming cetuximab resistance potentially by HER‐2/epidermal growth factor receptor dual inhibition.

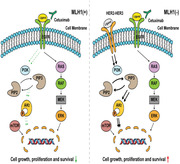

## 1 Introduction

Colorectal carcinoma (CRC) is one of the most common cancers worldwide. Colon cancer patients diagnosed with distant metastases have a poor 5 year survival rate at less than 10%.^[^ ^1^, ^2^ ^]^ Distant metastasis is the main reason for poor prognosis in CRC patients.^[^ ^3^, ^4^ ^]^ Cetuximab (CTX) is a monoclonal antibody that specifically binds to the epidermal growth factor receptor (EGFR) to inhibit the phosphorylation of receptor‐associated kinases and related signaling cascade.^[^ ^5^ ^]^ EGFR blockade can promote cell cycle arrest, tumor cell apoptosis, inhibit expression of vascular endothelial growth factor involved in invasion and metastasis, and prevent DNA repair after chemoradiotherapy.^[^ ^6^ ^]^ With the combination of targeted drugs such as CTX and chemotherapy, the median survival of patients with unresectable advanced colorectal cancer was extended from the previous 6–7 to 24–30 months, with improved quality of life.^[^ ^7^, ^8^ ^]^

However, more than half of CRC display heterogeneous genetic alterations leading to constitutive active EGFR signaling, which negatively affect response to EGFR monoclonal antibodies.^[^ ^9^ ^]^ Cetuximab is ineffective in CRC patients harboring KRAS or BRAF mutations. The response rate of patients with WT *KRAS/BRAF* to cetuximab is around 50%, and majority of patients who responded initially become refractory.^[^ ^10^, ^11^ ^]^ Primary resistance to anti‐EGFR antibodies in colorectal cancer is also caused by mutations in NRAS, as well as amplification of MET or ERBB2 (HER 2). Acquired resistance to cetuximab is an important clinical problem, and can be attributed to *RAS/RAF* gene mutation or amplification, *HER‐2* gene amplification, *MET* gene amplification, PIK3CA mutation, or low expression of PTEN.^[^ ^12^ ^]^ Strategies to overcome the resistance are being evaluated in clinical trials by combining anti‐EGFR with other targeted therapies.

Mismatch repair (MMR) plays an important role in maintaining genomic stability. Deficiency in MMR genes (dMMR), usually hMSH2 or hMLH1, promotes colon cancer development due to mutation or silencing.^[^ ^13^, ^14^ ^]^ dMMR tumors are defined by the accumulation of mutations, mostly insertions or deletions in short tandem repeats throughout the genome.^[^ ^15^ ^]^ These gene mutations participate in tumor initiation and progression as well as various DNA repair pathways. dMMR tumors are also linked to therapeutic resistance due to reduced DNA damage recognition and increased bypass of replicative lesions, leading to accumulation of mutations, genomic instability, and drug tolerance.^[^ ^16^, ^17^ ^]^ Previous studies provided substantial evidence that patients with dMMR colon cancers do not benefit from adjuvant FU/leucovorin.^[^ ^18^, ^19^ ^]^ In vitro studies have shown that MMR‐deficient cell lines display moderate levels of resistance to methylating agents and low level resistance to cisplatin^[^ ^20^ ^]^ but increased sensitivity to CPT.^[^ ^21^ ^]^ However, the role of MMR genes in cetuximab response remains largely unknown. Our study discovered mutL homolog 1 (MLH1) deficiency as a novel cetuximab resistance mechanism in CRC. Reduced MLH1 expression was correlated with poor prognosis in cetuximab‐treated patients. *MLH1* depletion led to activation of the Her‐2/phosphoinositide 3‐kinases (PI3K)/PKB protein kinase (AKT) signaling pathway and conferred resistance to cetuximab in vitro and in vivo. These results provide a better understanding of cetuximab response in CRC and potential way to overcome cetuximab resistance in microsatellite instability (MSI)‐high CRC.

## 2 Results

### 2.1 Reduced MLH1 Expression Is Correlated with Poor Prognosis in Cetuximab‐Treated CRC Patients

**Figure 1:**
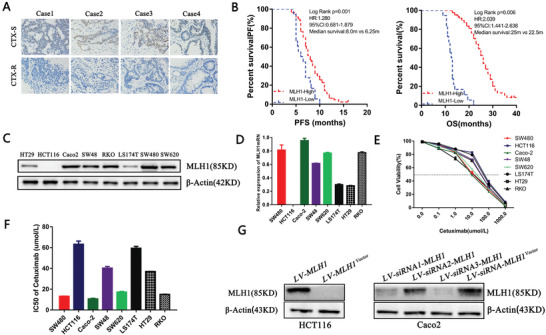
High MLH1 expression is associated with better prognosis in cetuximab‐treated colon cancer patients. A) MLH1 IHC staining in cetuximab‐sensitive and the cetuximab‐resistant CRCs (×200). Hematoxylin is the counterstain. B) Survival curves of PFS and OS of CTX‐treated CRC patients. C,D) MLH1 protein expression in eight CRC lines were examined by qRT‐PCR and western blot. Data are presented as the mean ± SD from three independent experiments. E,F) Eight CC cell lines were exposed to increasing concentrations of CTX from 0.1 × 10^−6^ to 1 × 10^−3^ m for 48 h to determine the IC50 values by CCK‐8 assay. Data are presented as the mean ± SD from three independent experiments. G) The LV‐MLH1 was stably transfected into HCT116 cells and three LV‐siRNA‐MLH1 were transfected into Caco‐2 cells. MLH1 protein expression was examined by western blot.

We first examined in CTX‐treated training cohort (*N* = 102, with 40 months follow up) of MLH1 protein expression by immunohistochemistry (IHC) MLH1 protein was mainly located in the nucleus of cancer cells (**Figure** **1**A) and its reduction was found to be associated with poor prognosis. Based on the IHC scoring standard, 82.4% (84/102) and 17.6% (18/102) of tumors were defined as showing high and low MLH1 protein expression, respectively (**Table** **1**). The median progression‐free survival (PFS) and overall survival (OS) in the high MLH1 and low MLH1 expression group were 8.0 months versus 6.25 months (95% confidence interval (CI), 0.681–1.879) and 25.0 months versus 22.5 months (95% CI, 1.441–2.638), respectively (*p* = 0.006 and *p* = 0.001) (Figure 1B). A multivariable Cox proportional hazards model revealed that MLH1 expression in CRC is an independent prognostic factor for PFS (hazard ratio [HR] = 2.030, *p* = 0.034) (**Table** **2**).

**Table 1:** Relationship between MLH1 expression and the clinical pathological characteristics in training cohort and validation cohort of CRC

Clinical pathological indexes		Training cohort		*p*		Validation cohort	
		MLH1(+) (*N*) [%]	MLH1(−) (*N*) [%]		MLH1(+) (*N*) [%]	MLH1(−) (*N*) [%]	*p*
Age	≤51	36 (35.3)	10 (9.8)	0.326	26 (31.0)	11 (13.1)	0.376
	>51	48 (47.1)	8 (7.8)		41 (48.8)	6 (7.1)	
Sex	Male	42 (41.2)	12 (11.8)	0.199	35 (41.7)	11 (13.1)	0.184
	Female	42 (41.2)	6 (5.8)		32 (38.1)	6 (7.1)	
Tumor size	≤5 cm	63 (61.8)	9 (8.8)	0.035	50 (59.5)	8 (9.5)	0.045
	>5 cm	21 (20.6)	9 (8.8)		17 (20.3)	9 (10.7)	
Tumor differentiation	Well	59 (57.8)	10 (9.8)	0.227	19 (22.6)	7 (8.3)	0.277
	Poor	25 (24.5)	8 (7.9)		48 (57.2)	10 (11.9)	
Tumor invasion	T1+T2	32 (31.4)	4 (3.9)	0.201	22 (26.2)	5 (6.0)	0.281
	T3+T4	52 (51.0)	14 (13.7)		45 (53.6)	12 (14.4)	
ECOG score	0–1	62 (60.8)	10 (9.8)	0.123	54 (64.3)	9 (10.7)	0.128
	≥2	22 (21.5)	8 (7.9)		13 (15.5)	8 (9.5)	

**Table 2:** Multivariate analysis by a Cox proportional hazards regression model in training cohort and validation cohort

Variable	Training cohort				Validation cohort			
	PFS		OS		PFS		OS	
	HR (95% CI)	*p*	HR (95% CI)	*p*	HR (95% CI)	*p*	HR (95% CI)	*p*
Age (≤51 vs >51)	0.943 (0.616–1.443)	0.787	0.769 (0.485–1.217)	0.262	0.843 (0.626–1.643)	0.645	0.779 (0.465–1.116)	0.162
Tumor size (≤5 cm vs >5 cm)	1.467 (0.940–2.289)	0.092	1.435 (0.932–2.209)	0.101	1.327 (0.940–2.289)	0.112	1.215 (0.936–2.149)	0.121
Tumor differentiation (well vs poor)	1.093 (0.680–1.759)	0.713	1.001 (0.627–1.600)	0.995	1.113 (0.760–1.723)	0.523	1.201 (0.727–1.530)	0.975
Tumor invasion (T1+T2 vs T3+T4)	0.992 (0.612–1.608)	0.974	1.031 (0.598–1.775)	0.913	0.872 (0.512–1.408)	0.654	1.121 (0.628–1.425)	0.543
ECGO (0–1 vs ≥2)	0.89 (0.556–1.455)	0.666	0.976 (0.603–1.578)	0.921	0.779 (0.456–1.235)	0.676	0.864 (0.564–1.238)	0.771
Chemotherapy (FOLFOX vs FOLFORI)	1.140 (0.681–1.910)	0.618	1.551 (0.885–2.719)	0.126	1.121 (0.651–1.620)	0.652	1.421 (0.565–2.239)	0.426
MLH1 expression (high vs low)	2.030 (1.057–3.901)	0.034	1.122 (0.596–2.112)	0.721	2.110 (1.057–3.101)	0.021	1.422 (0.796–2.021)	0.821

### 2.2 MLH1 Expression Levels Are Associated with Cetuximab Sensitivities in CRC Cells

To explore the association between MLH1 expression and CTX sensitivity in vitro, we determined the MLH1 protein and mRNA levels in eight CRC cell lines. High MLH1 expression was found in Caco‐2, SW480, SW620, and RKO cells, while low MLH1 expression was found in LS174T and HT29 cells. HCT116 cells have MLH1 deletion and no MLH1 expression (Figure 1C,D). These eight CRC lines were exposed to increasing concentrations of CTX from 0.1 × 10^−6^ to 1 × 10^−3^ m for 48 h to determine the IC50 values. Caco‐2 cells (IC50 = 9.21 × 10^−6^ m) exhibited the greatest CTX sensitivity, while HCT116 cells (IC50 = 62.17 × 10^−6^ m) were the most resistant (Figure 1E,F). Caco‐2 cells and HCT116 cells were chosen for subsequent in vitro and in vivo experiments. The lentivirus (LV)‐MLH1 was stably transfected into HCT116 cells to upregulate expression of MLH1 while three LV‐small interfering RNA (siRNA)‐MLH1 were transfected into Caco‐2 cells to knockdown expression of MLH1 and LV‐siRNA3‐MLH1 with the maximum knockdown efficiency (Figure 1G).

### 2.3 CRC Cells with MLH1 Deletion Are Resistant to Cetuximab In Vitro

**Figure 2:**
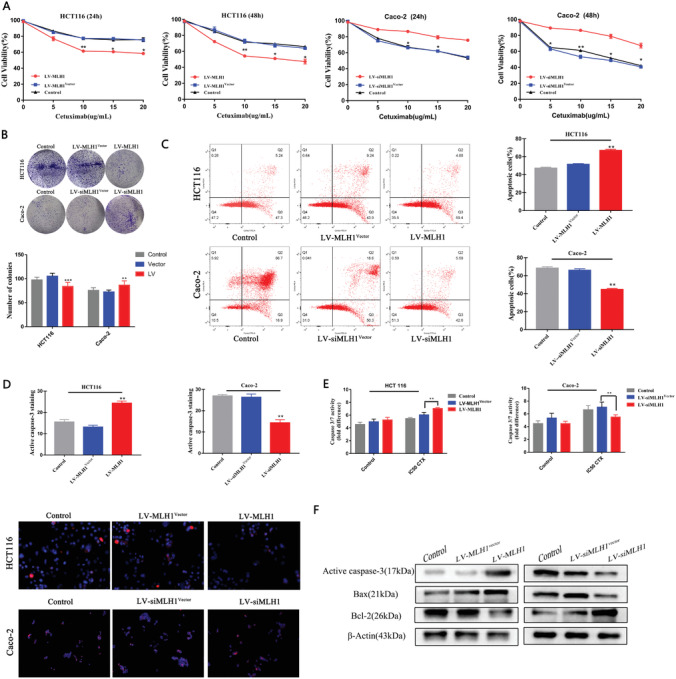
*MLH1* loss increases CTX resistance in vitro. HCT 116 and Caco‐2 cells with MLH1 overexpression or knockdown, or control vector. All cells received CTX treatment at the respective IC50 concentration for 48 h unless otherwise indicated. A) Cell viability was determined by the CCK‐8 assay. *n* = 3. B) Colony‐formation visualized on day 14 (*p* < 0.05) (*p* < 0.01). C) Apoptotic cells were analyzed by flow cytometry (*p* < 0.01). D) Active caspase‐3 was analyzed by immunostaining (red) (*p* < 0.01). E) Caspase‐3 and caspase‐7 activities were measured by the Caspase‐Glo 3/7 Assay (*p* < 0.01). F) The levels of active caspase‐3, Bax, and Bcl‐2 were examined by western blot. Data are presented as the mean ± SD from three independent experiments.

Differential viability of HCT116 cells and Caco‐2 cells to cetuximab treatment (5, 10, 15, and 20 µg mL^−1^) was confirmed at 24 and 48 h after 2% fetal bovine serum (FBS) overnight starvation. Caco‐2 cells also showed reduced colony‐formation ability compared with HCT 116 cells (*p* < 0.05; **Figure** **2**A,B). Overexpression of MLH1 increased CTX sensitivity in HCT 116 cells, while knockdown of MLH1 decreases CTX sensitivity of Caco‐2 cells. Compared to HCT 116 cells, Caco‐2 cells showed elevated apoptosis and activation of caspase‐3/‐7 (Figure 2C–E; *p* < 0.01). Western blot demonstrated significantly reduced expression of the antiapoptotic protein Bcl‐2 and increased expression of the proapoptotic proteins Bax and active caspase‐3 in Caco‐2 cells (Figure 2F). Overexpression of MLH1 increased apoptosis, caspase‐3/‐7 activation, and Bax expression and decreased Bcl‐2 expression in HCT 116 cells. Knockdown of MLH1 had opposite effects in Caco‐2 cells (Figure 2D–F).

### 2.4 MLH1 Deletion in CRC Cells Leads to Cetuximab Resistance In Vivo

**Figure 3:**
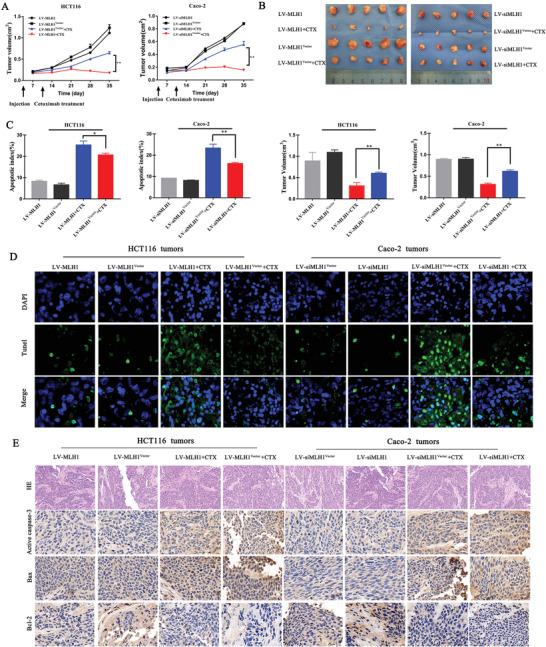
MLH1 mediates CRC sensitivity to CTX in vivo. A) CRC cells with different MLH1 expression levels were implanted subcutaneously into nude mice. Tumor growth was examined by measuring the tumor volume after 3 weeks of treatment with cetuximab (10 mg kg^−1^) (every 2 days; *n* = 8). Line graph showing the tumor volume (mm^3^) from day 0 to day 34. Error bars represent the mean ± SD from 8 mice. For statistical analysis, Student's *t*‐test (two‐sided, paired) was used. ^*^ *p* < 0.05 and ^**^ *p* < 0.01. B) Five weeks after subcutaneous implantation, the average tumor volumes in each group were calculated using the following formula: *V* (mm^3^) = (*L* × *W*2) × 0.5 (*L*: tumor length; *W*: width). C,D) TUNEL assay for CC apoptosis detection in subcutaneously implanted tumors with different MLH1 expression levels (original magnification: 200). E) Immunohistochemistry staining for apoptosis‐related protein expression in subcutaneous tumors (original magnification: 400). Data are shown as the mean ± SD from three independent experiments. ^*^ *p* < 0.05, ^**^ *p* < 0.01, and ^***^ *p* < 0.001.

To investigate the role of MLH1 in CRC growth and cetuximab sensitivity in vivo, HCT116 and Caco‐2 cells with different MLH1 expression levels were subcutaneously injected into nude mice with or without cetuximab treatment. MLH1 expression affected CTX response after 3 weeks either HCT116 or Caco‐2 xenografts. MLH1‐high HCT116 and Caco‐2 tumors grew at a significantly lower rate than those with no or lower expression (**Figure** **3**A,B). The volume of HCT116 xenografts in LV‐MLH1 CTX group was smaller than that of the LV‐Control CTX group (0.58 ± 0.01 cm^3^ vs 0.28 ± 0.02 cm^3^, *p* < 0.01). The volume of Caco‐2 xenografts in LV‐siMLH1 CTX group was larger than that of the LV‐Control CTX group (0.54 ± 0.03 cm^3^ vs 0.24 ± 0.02 cm^3^, *p* < 0.01). Terminal deoxynucleotidyl transferase (TdT) dUTP nick‐endlabeling (TUNEL) assay was performed to assess tumor cell apoptosis. Apoptosis rates of HCT116 cells in the LV‐Control, LV‐MLH1, LV‐Control CTX, and LV‐MLH1 CTX groups were 9.33 ± 0.23%, 8.21 ± 0.38%, 23.23 ± 0.88%, and 18.00 ± 0.92%, respectively (Figure 3C,D; *p* < 0.05). In contrast, MLH1 knockdown attenuated CTX‐induced apoptosis in Caco‐2 xenografts. Apoptosis rates of Caco‐2 cells in the LV‐siMLH1, LV‐siMLH1^Vector^, LV‐siMLH1 CTX, and LV‐siMLH1^vector^ CTX groups were 9.33 ± 0.33%, 8.33 ± 0.33%, 21.00 ± 1.53%, and 17.67 ± 1.20%, respectively (Figure 3C,D; *p* < 0.01). Higher levels of Bax and active caspase‐3 and lower Bcl‐2 were observed in the MLH1‐high compared to MLH1‐low group (Figure 3E). These data were consistent with those obtained in vitro (Figure 2) and support that MLH1 reduction leads to CTX resistance and inhibits CTX‐induced apoptosis in vivo.

### 2.5 Her‐2/PI3K/AKT Signaling Plays an Important Role in MLH1‐Related CTX Sensitivity in CRC

**Figure 4:**
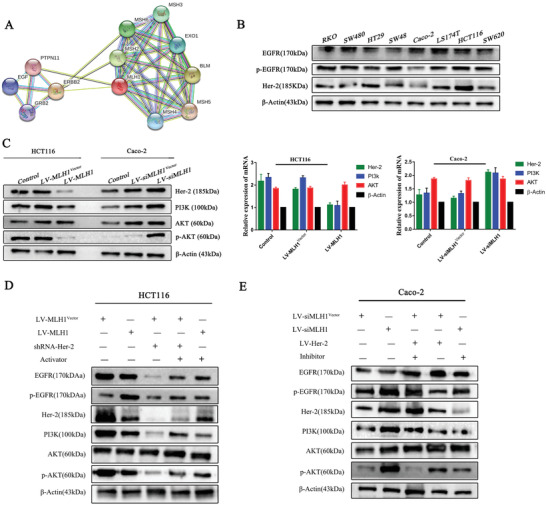
Signaling pathways involved in the MLH1‐mediated cetuximab resistance in CRC cells. A) Bioinformatics analysis: The Gene MANIA database and the STRING database were searched to make predictions. B) Western blotting was performed to detect levels of EGFR, p‐EGFR, and Her‐2 in eight CRC cell lines. *β*‐Actin was used as a loading control. C) MLH1 was overexpressed in HCT116 cells transfected with LV‐MLH1 and was silenced in Caco‐2 cells transfected with LV‐siMLH1. The levels of Her‐2, PI3k, AKT, and p‐AKT were detected by western blotting. *β*‐Actin was used as a loading control. Data are expressed as the means of three independent experiments. D,E) HCT116 cells overexpressing MLH1 were treated with an activator of the PI3K/AKT signaling pathway, while MLH1 negative control HCT116 cells were transfected with shRNA‐Her‐2. MLH1‐knockdown Caco‐2 cells were treated with an inhibitor of the PI3K/AKT signaling pathway, while MLH1 negative control Caco‐2 cells were transfected with LV‐Her‐2. The levels of Her‐2, PI3k, AKT, and p‐AKT were detected by western blotting. *β*‐Actin was used as a loading control.

To explore the potential mechanisms of MLH1‐mediated cetuximab sensitivity in CRC, we analyzed MLH1‐related signaling pathways using Gene MANIA database and the STRING database. Her‐2/PI3K/AKT signaling was nominated as a candidate pathway (**Figure** **4**A). Western blotting was performed to detect the levels of EGFR, p‐EGFR, and Her‐2 in eight CRC cell lines. Higher expression of Her‐2 and p‐EGFR was found in cell lines with lower MLH1 expression (Figure 4B). Overexpression of MLH1 decreased the levels of HER‐2, PI3K, and p‐AKT (S473) in HCT 116 cells, while knockdown of MLH1 increased these markers in Caco‐2 cells (Figure 4C).

**Figure 5:**
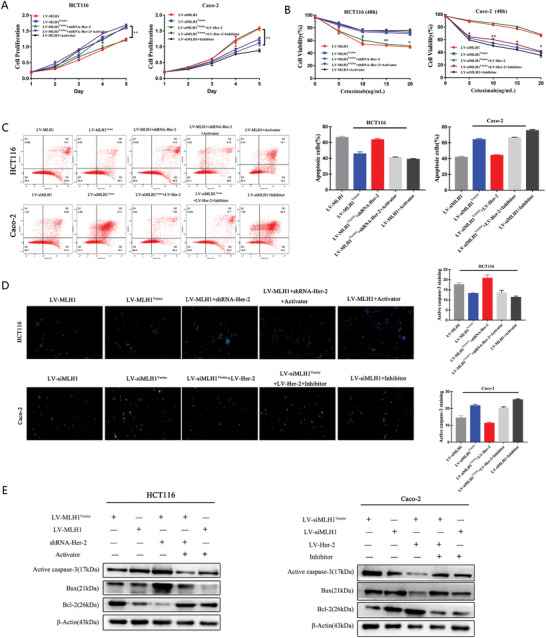
MLH1 depletion enhances CRC resistance to CTX by inducing the Her‐2/PI3K/AKT signaling pathway in vivo. HCT116 cells overexpressing MLH1 were treated with an activator of the PI3K/AKT signaling pathway, while MLH1 negative control HCT116 cells were transfected with shRNA‐Her‐2. MLH1‐knockdown Caco‐2 cells were treated with an inhibitor of the PI3K/AKT signaling pathway, while MLH1 negative control Caco‐2 cells were transfected with LV‐Her‐2. All CRC cells received CTX treatment for 48 h at the respective IC50 concentration unless otherwise indicated. A) CCK‐8 analysis of HCT116 cell proliferation with the above‐mentioned treatment. CRC cells were treated with the indicated treatments. B) Cells were treated with increasing concentrations of cetuximab (5, 10, 15, and 20 µg mL^−1^) for 48 h, and cell viability was determined by the CCK‐8 assay. C) FACS analysis of cell apoptosis in HCT116 and Caco‐2 cells with the above‐mentioned treatment. D) Caspase‐3 activation after CTX treatment, as indicated by green fluorescent staining. E) Expression of proapoptotic proteins (Bax and active caspase‐3) and an antiapoptotic protein (Bcl‐2) in CTX‐treated CRC cells. Data are presented as the mean ± SD from three independent experiments. ^*^ *p* < 0.05, ^**^ *p* < 0.01, and ^***^ *p* < 0.001.

We knocked down Her‐2 using shRNA in HCT116 cells (shRNA‐Her‐2) and examined Her‐2/PI3K/AKT signaling and CTX sensitivity. Knockdown of Her‐2 reduced levels of PI3K and p‐AKT. However, the above phenomenon was reversed when a pathway activator SC79 (S7863) was administered (Figure 4D). We transfected LV‐Her‐2 into Caco‐2 cells to examine Her‐2/PI3K/AKT‐related markers and CTX sensitivity. The results showed activation of Her‐2/PI3K/AKT signaling leads to CTX resistance, while blocking the pathway with an inhibitor Piperlongumine (S7551), increased drug sensitivity (Figure 4E). CCK‐8 assays showed that the drug sensitivity was dramatically improved (**Figure** **5**A,B). Apoptosis analysis showed that knockdown of HER‐2 increases cell apoptosis after cetuximab treatment (Figure 5C). shRNA‐Her‐2 transfection in HCT116 cells led to increased caspase‐3 activation levels (Figure 5D). Western blot confirmed higher Bax and active caspase‐3 and lower Bcl‐2 expression (Figure 5E)

### 2.6 MLH1‐Regulated CTX Resistance via the Her‐2/PI3K/AKT Signaling Pathway in the Validation Cohort and In Vivo

**Figure 6:**
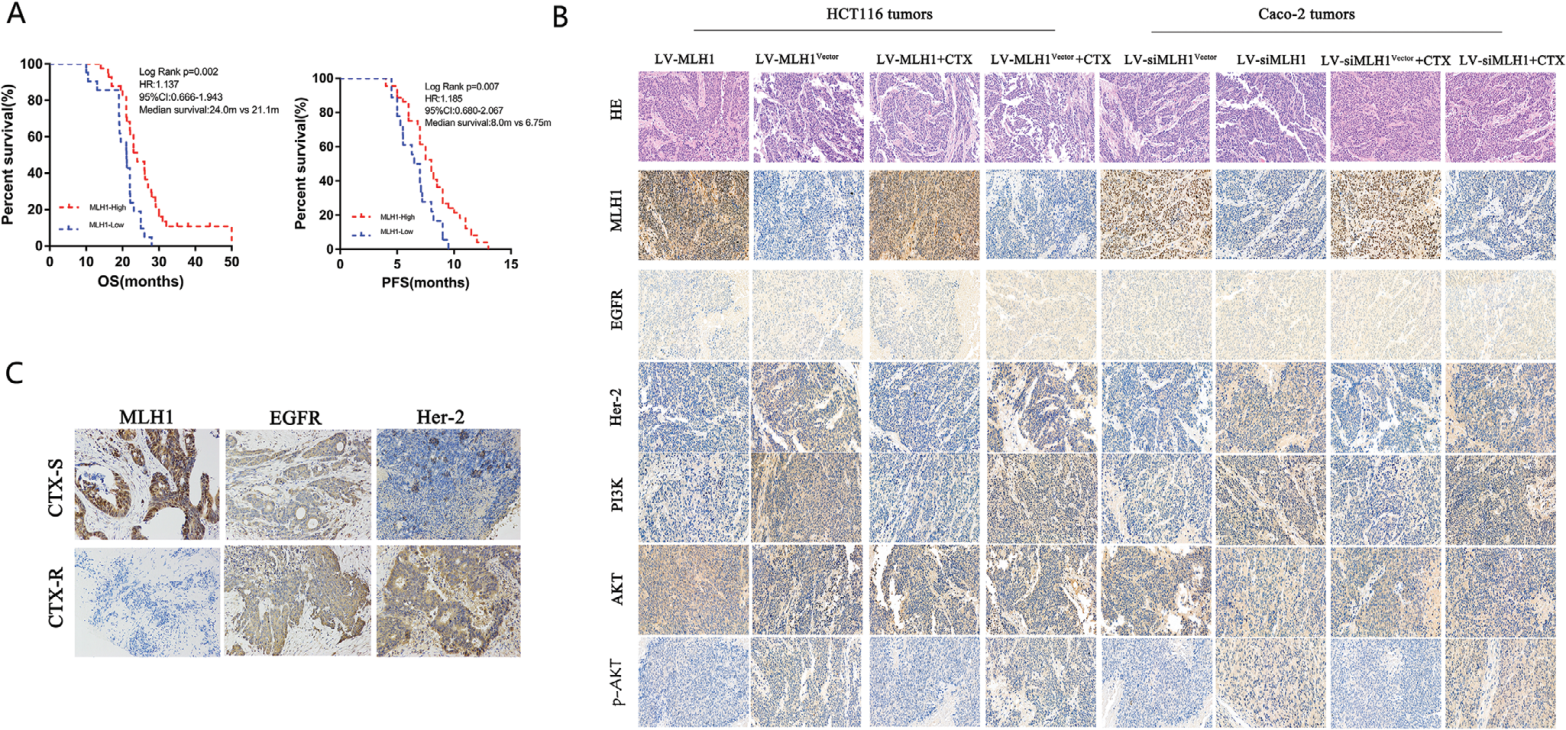
MLH1 depletion enhances CRC resistance to CTX by inducing the Her‐2/PI3K/AKT signaling pathway in the validation cohort and in vivo. A) Survival curves, which were evaluated by the log‐rank test, indicated the PFS and OS of CRC patients with high or low MLH1 expression in the validation cohort (*n* = 84). B) Expression of MLH1, EGFR, Her‐2, PI3k, AKT, and p‐AKT in tumors formed from CRC cells that were subcutaneously injected into nude mice as detected by hematoxylin and eosin (HE) and IHC staining. C) IHC staining for MLH1, EGFR, and Her‐2 protein expression in CRC tissues (T) from the cetuximab‐sensitive group and the cetuximab‐resistant group (×200).

We then further analyzed MLH1‐HER‐2 signaling in CTX response in CRC patients using a validation cohort. The rates of low and high MLH1 expression in CRC tissue were 79.8% (67/84) and 20.2% (17/84), respectively. The median PFS and OS were 8.0 months versus 6.75 months (95% CI, 0.680–2.067) and 24.0 months versus 21.1 months (95% CI, 0.666–1.943) in the high MLH1 expression group and the low MLH1 expression group, respectively, with statistically significant differences (*p* = 0.002 and *p* = 0.007) (**Figure** **6**A). A multivariate Cox proportional hazards model constructed using the training cohort showed that MLH1 expression was an independent prognostic factor for PFS (HR = 2.110, *p* = 0.02; Table 2). Results of IHC assay performed in subcutaneous tumors were consistent with western blot findings in CRC cells. Higher Her‐2, PI3K, and p‐AKT expression levels were observed in the MLH1‐low group than in the MLH1 high group, which were observed in both the HCT116 tumors and Caco‐2 tumors (Figure 6B). Additionally, we examined MLH1 and Her‐2 expression in cetuximab‐sensitive or cetuximab‐resistant CRC tissues. The IHC results revealed higher levels of Her‐2 in cetuximab‐resistant CRC tissues, compared to cetuximab‐sensitive CRC tissues (Figure 6C).

## 3 Discussion

**Figure 7:**
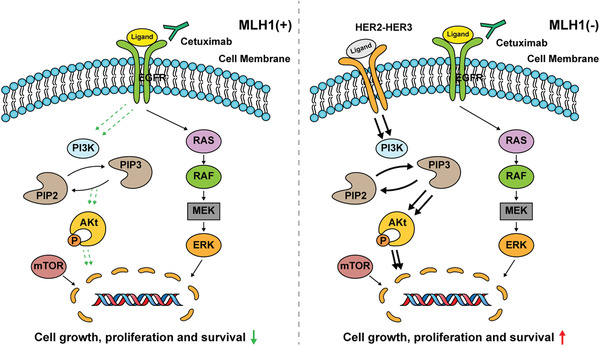
Proposed working model. In MLH1(+) colon cancer cells, anti‐EGFR antibody cetuximab binds to the EGFR and promotes the internalization and degradation of the EGFR, abrogating its downstream PI3K/AKT signaling cascades, thus leading to inhibition of cancer growth and progression. However, in MLH1(−) colon cancer cells, HER2/3 homodimerization or heterodimerization leads to PI3K/AKT activation and increased cell growth and survival, resulting in cetuximab resistance.

Cetuximab was the first FDA‐approved anti‐EGFR antibody for CRC therapy, and has been used in combination with standard chemotherapy or radiotherapy in locally advanced, metastatic, and recurrent CRC.^[^ ^22^, ^23^ ^]^ However, cetuximab resistance remains a major clinical challenge. Cetuximab targets EGFR on the cell membrane and increases receptor internalization and degradation to enhance antibody‐dependent cell‐mediated cytotoxicity in addition to inhibiting RTK signaling.^[^ ^24^ ^]^ Genetic activation of KRAS, NRAS, HER2, and MET or upregulation of EGFR, HER‐2, and HER‐3 IGF1R can lead to acquired resistance to cetuximab.^[^ ^25^, ^26^, ^27^ ^]^ Acquired mutations in RAS/RAF, HER‐2, and PI3K have also been identified in circulation tumor DNA (ctDNA) in CTX‐treated mCRC patients.^[^ ^28^, ^29^ ^]^ Our study identified loss of MLH1 as a novel CTX resistance mechanism through activation of HER‐2/PI3K/AKT signaling (**Figure** **7**A).

MMR gene is mutated in 87% of patients with Lynch syndrome and was predominantly deficient in hMLH1 and hMSH.^[^ ^30^ ^]^ In sporadic CRC, 10–20% of patients are dMMR, and approximately 95% was due to methylation of the MLH1 gene promoter region.^[^ ^31^, ^32^ ^]^ A growing number of studies have shown that loss of MMR gene function is associated with chemotherapy resistance, and promotes clonal expansion in colon cancer and breast cancer.^[^ ^33^ ^]^ An earlier study indicated preferential activation of PI3K/AKT rather than ERK/MPAK signaling in MSI CRCs.^[^ ^34^ ^]^ Intriguingly, BRAF WT MSI CRC was characterized by a high frequency of recurrent Her‐2 (ERBB2) mutations, including hot spots L755S and V842I in sporadic cases.^[^ ^35^, ^36^ ^]^ Pathogenic MMR germline mutations were also associated with HER‐2‐mutated MSI CRC. These findings support that MLH1 loss increases Her‐2/PI3K/AKT signaling, while the underlying mechanism remains unclear. It would be interesting to determine loss of other MMR gene might lead to CTX resistance via similar mechanism in intrinsic or acquired settings.

Understanding molecular mechanisms underlying therapeutic resistance is critical to develop more effective therapies. HER‐2 acts as a mediator of the EGFR bypass signaling pathway, and HER‐2 gene amplification stimulates downstream the RAF‐MEK‐ERK and PI3K‐PTEN‐AKT signaling pathways, leading to increases proliferation and survival.^[^ ^37^, ^38^ ^]^ HER‐2 mutation or amplification in treatment‐naive mCRC is rare and their impacts on the clinical features is not substantial.^[^ ^39^, ^40^, ^41^ ^]^ Interestingly, cetuximab‐resistance in mCRC often exhibited MLH1 deletion and HER‐2 amplification or mutations in the coding sequence. Our findings suggest a selective dependence of MSI CRC on HER‐2/PI3K, which might be effectively targeted by dual inhibition of EGFR with HER‐2 or PI3K inhibitor (pictilisib). This phenomenon is consistent with the well‐established feedback activation of PI3K/AKT as a major resistance mechanism in EGFR/mitogen‐activated protein kinase (MAPK)‐targeted therapies in solid tumors including CRC and breast cancer,^[^ ^42^ ^]^ which is also associated with increased aggressiveness.^[^ ^43^, ^44^ ^]^ Therefore, it might be interesting to evaluate if EGFR combination with HER‐2 or PI3K inhibitors offers more durable response in MSI KRAS/BRAF wildtype CRCs.

In summary, our findings demonstrated MLH1 loss as a novel mechanism of cetuximab resistance in CRC through activation of Her‐2/PI3K/AKT signaling. These findings revealed an interesting link between DNA damage response and growth factor signaling, and potential combination therapy to treat MSI and HER‐2 high mCRC.

## 4 Experimental Section

##### Patients and Follow Up

Two independent cohorts of 186 CRC patients were enrolled in this study. In the training cohort (paraffin‐embedded tissues, *n* = 102), tumor tissues were collected from advanced CRC subjects between January 2010 and March 2013 in Xiangya Hospital Central South University, Changsha, Hunan, China. The CRC cases in the validation cohort (*n* = 84) were collected between April 2013 and May 2016. Informed consent was obtained from the recruited patients, and the study protocols were approved by the Ethics Committees of the Xiangya hospitals.

All patients enrolled were histologically diagnosed as unresectable CRC with no prior treatment were included, as were those CRC patients who had received postoperative adjuvant chemotherapy 1 year before assignment. Included patients met the following criteria: 1) pathological diagnosis of colon adenocarcinoma; 2) clinical diagnosis of advanced colorectal cancer; 3) adequate organ and marrow function, with previous treatment with anticancer agents or herbal treatments completed ≤4 weeks before the random assignment; 4) receipt of first‐line cetuximab + chemotherapy with FOLFOX or FOLFIRI; and 5) no family history of genetic disease, with no serious or fatal diseases of the liver, lung, kidney, or other organs. Exclusion criteria were as follows: less than 4 cycles of chemotherapy.

The follow‐up (terminated on April 30, 2019) period was defined as the interval between the date of random assignment and that of the patient's death or the last follow up. The primary study endpoints were OS, which was defined as the time from the date of the first cycle of therapy to the date of death from any cause, and first‐line PFS, which was defined as the time from the initial date of therapy to tumor progression, death from any cause, or the last follow up before the initiation of second‐line therapy. The study was approved by the Ethics Committee of the institution.

Every 2 months, the response to treatment was evaluated by computed tomography scans according to the Response Evaluation Criteria in Solid Tumors (RECIST, version 1.1).

##### Reagents and Antibodies

The PI3K/AKT Inhibitor Piperlongumine (S7551) and AKT activator SC79 (S7863) were purchased from Selleck‐chem (Houston, TX, USA). Reagents were reconstituted and stored according to the manufacturer's instructions. Antibodies against MLH1 (ab124715), EGFR (#4108), p‐EGFR (ab56416), Her‐2 (BECN1, ab207612), PI3K (ab213521), AKT (#4668), Bcl‐2 (#4223), Bax (ab51520), active caspase‐3 (ab15580), and GAPDH (ab181602) were purchased from Cell Signaling Technology (Beverly, MA, USA) and Abcam (Cambridge, UK).

##### Cell Lines and Cell Culture

Human colon cancer cell lines RKO, HCT116, SW48, HT29, LS174T, Caco‐2, SW480, and SW620 were obtained from the Institutes of Biomedical Sciences (IBS, Shanghai, China). Among them, microsatellite stable (MSS) cell lines were Caco‐2, SW480, HT29, and SW620, and MSI cell lines were RKO, HCT116, LS174T, and SW48. Cells were cultured in DMEM (Gibco, Grand Island, NY) or RPMI‐1640 (HyClone, Logan, UT) supplemented with 10% FBS.

##### Immunohistochemistry

Xenograft tumors were fixed in 10% formalin, dehydrated, and embedded in paraffin, and cut into 4 µm sections. After undergoing dewaxing and hydration, antigen retrieval, and blocking of endogenous peroxidase activity, the slides were then incubated with primary antibodies at 4 °C overnight, using the following primary antibodies: MLH1 (1:5000), EGFR (1:2000), p‐EGFR (1:2000), Her‐2 (1:2000), PI3K (1:2000), AKT (1:2000), and p‐AKT (1:2000). Briefly, the results were judged by an independent pathologist according to the percentage of positive‐staining cells and staining intensity based on the following scoring method: The staining intensity was evaluated on a 0–3 scale (0, negative; 1, weakly positive; 2, moderately positive; 3, strongly positive), while percentage of positive cells were evaluated on a 0–4 scale (0, negative; 1, positive in 1–25%; 2, positive in 26–50%; 3, positive in 51–75%; 4, positive in 76–100%). The score of percentage and intensity were multiplied and the results were classified into positive (the total scores ≥4) and negative (the total scores <4).

##### Cell Viability Assay

Cell viability was determined with a Cell Counting Kit‐8 (CCK‐8, CK04‐1000T, Dojindo Molecular Technologies, Inc., Tokyo, Japan). Briefly, cells (2.0 × 10^3^ per well) were pipetted into a 96‐well plate subjected to various indicated treatments, 10 µL CCK‐8 was added to each well for 2 h, and then the absorbance at 450 nm was measured. Each assay was performed in triplicate. Dose curves after cetuximab administration were plotted to calculate the 50% growth inhibition (IC50) values.

##### Quantitative Real‐Time Reverse Transcription Polymerase Chain Reaction (qRT‐PCR)

Total RNA was extracted using TRIzol Reagent (Invitrogen, Carlsbad, CA), and amplification of the cDNA (1 µg per sample) was performed using the Prime Script Kit (TaKaRa Bio Inc., Otsu, Japan) according to the manufacturer's indication. Real‐time PCR was performed in triplicate by SYBR Green fluorescent‐based assay (638320, TaKaRa Bio Inc.) on a ViiATM7 RT‐PCR system (Applied Biosystems, Carlsbad, CA). The primers for real‐time PCR were as follows: MLH1: forward: 5′‐CTCCAAGATGAGGCTGTAGGAA‐3′; reverse: 5′‐CCTATGAGATGGAAGGCAAGA‐3′; GAPDH forward: 5′‐CTGGGCACTGAGCACC‐3′; reverse: 5′‐AAGTGGTCGTTGAGGGCAATG‐3′; Her‐2 forward: 5′‐GATCCCCTGATAGACACCAACCGCTCTTCAAGAGAGAGCGGTTGGTGTCTATCATTTTTGGAAA‐3′; reverse: 5′‐AGCTTTTCCAAAAATGATAGACACCAACCGCTCTCTCTTGAAGAGCGGTTGGTGTCTATCAGGG‐3′; PI3K forward: 5′‐TGCTATGCCTGCTCTGTAGTGGT‐3′; reverse: 5′‐GTGTGACATTGAGGGAGTCGTTG‐3′; and AKT forward: 5′‐GTGCTGGAGGACAATGACTACGG‐3′; reverse: 5′‐AGCAGCCCTGAAAGCAAGGA‐3′. Relative mRNA expression levels were calculated by the 2^−(ΔΔCt)^ method and were normalized to the internal control (GAPDH), ΔCt = Ct (targeting gene) − Ct (GAPDH), and ΔΔCt = ΔCt (treated) – ΔCt (control).

##### Western Blot

Total protein was extracted in radioimmunoprecipitation assay (RIPA) lysis buffer (P0013B, Beyotime, Shanghai, China). Protein concentration was determined using the BCA protein assay kit (Beyotime Biotechnology, Shanghai, China). Protein samples were separated by 10% SDS‐PAGE and then transferred onto 0.2 µm Polyvinylidene fluoride (PVDF) membranes (Millipore, Bedford, MA) under a constant 300 mA. Then, the membrane was incubated with the primary antibodies at 4 °C overnight after being blocked in TBST (Tris‐buffered saline (TBS) with 0.5% Tween) containing 5% skim milk at 37 °C for 1 h. After incubating with an Horseradish Peroxidase (HRP)‐conjugated secondary antibody for 1 h at 37 °C,the membrane was washed in TBST and prepared for signal detection. The signals were automatically visualized using the ChemiDoc XRS+ System (Bio‐Rad, Hercules, CA) and quantitatively analyzed with Image Lab software (Bio‐Rad). *β*‐Actin protein expression was used as the internal control.

##### Colony‐Formation Assays

Cells were seeded in 6‐well plate (Costar, Cambridge, MA) at a density of 1 × 10^3^ per well and then cultured with various treatments for 2 weeks’ colonies formation. Before colonies counting,the cells were fixed with methanol for 15 min and stained with 0.1% crystal violet for 20 min at room temperature. Colonies containing ≥50 cells were manually counted under a microscope. Each assay was performed in triplicate.

##### Apoptosis Assay

The apoptosis was analyzed by flow cytometry on a FACS Canto II flow cytometer (BD Biosciences). Annexin V‐FITC Apoptosis Detection Kit I (catalog# 556547, BD Pharmingen, USA) was applied to assess apoptosis according to the manufacturer's instructions. Briefly, cells resuspended in 1× binding buffer at a concentration of 1 × 10^6^ cells mL^−1^ were added to a tube containing both Annexin V‐FITC reagent (5 µL) and a PI reagent (5 µL), incubating for 15 min at room temperature in the dark before analysis.

##### Immunofluorescence (IF) Staining and Confocal Microscopy Analysis

The treated cells were washed with ice‐cold phosphate‐buffered saline (PBS, pH 7.4) and fixed with 4% paraformaldehyde. The cells were then permeabilized with 1% Triton X100 + 0.1% SDS, blocked in 2% bovine serum albumin for 30 min prior to incubation with the primary antibodies against active caspase‐3 (Abcam, Cambridge, UK; 1:200 dilution) overnight at 4 °C, and subsequently incubated with the corresponding Alexa Fluor 546‐ and Alexa Fluor 488‐conjugated secondary antibodies (Life Technologies, Waltham, MA, 1:2000 dilution). Images were captured using a TCS SP8 confocal microscope (Leica, Wetzlar, Germany).

##### Construction of the MLH1 Overexpression Lentivirus and the Transfection of HCT116 Cells

The lentivirus vector (GV18549) cloned with PCR product covering the MLH1 open reading frame was constructed by GeneChem. The lentivirus was stably transfected into HCT116 cells, and the upregulated expression of MLH1 in the pool was confirmed by western blotting.

##### Construction of MLH1 siRNA Lentivirus and Infection in Caco‐2 Cells

An MLH1 RNA interference (RNAi) lentiviral vector (GV52866‐si‐MLH1) was constructed by GeneChem. The lentivirus was stably transfected into Caco‐2 cells, and the siRNA sequences with the maximum knockdown efficiency (78.7%) were selected for further stable MLH1 silencing in Caco‐2 cells for animal experiments.

##### Her‐2‐shRNA Plasmid and Transfections

HCT116 cells were transfected with a Her‐2‐shRNA plasmid (GeneChem) using Lipofectamine 3000 (Invitrogen, Carlsbad, CA), followed by selection of positive colonies with puromycin (5 mg mL^−1^, Invitrogen).

##### In Vivo Xenograft Studies

All mice were treated humanely, and the protocols for treating animals had been approved by the Medical Experimental Animal Care Commission of Central South University. Stable pooled Caco‐2 cells infected with LV‐siRNA‐MLH1 lentivirus and HCT116 cells infected with LV‐MLH1 lentivirus were subcutaneously injected into the right flank regions of male BALB/c mice (4 weeks of age, 5 × 10^6^ per mouse). Ten days later, six mice from each experimental and control group received intraperitoneal injections of cetuximab (10 mg kg^−1^, every 2 days for 3 weeks). The tumor growth was monitored 1 week after implantation, and all mice were sacrificed after 5 weeks. The final tumor sizes were removed and calculated. IHC staining was performed on cell proliferation and apoptosis markers (Bcl‐2, Bax, and Caspase‐3) and Her‐2/PI3K/AKT signaling pathway‐related markers. According to the manufacturer's protocol, TUNEL assay kit (Roche, Basel, Switzerland) was used to detect colon cell apoptosis. Fluorescence images were obtained using a Carl Zeiss confocal microscope (Weimar, Germany).

##### Statistical Analysis

Statistical analysis was performed by GraphPad Prism 7.0 software. All data were presented as the mean ± SD, and two‐tailed unpaired or paired Student's *t*‐test and ANOVA (Dunnett's or Fisher's Least Significant Difference(LSD) posthoc test) were used accordingly. Survival curves were calculated using the Kaplan–Meier method and the log‐rank test. The Cox proportional hazards regression model was used to identify the predictive factors that independently affected the prognosis of colon cancer. All tests were two‐sided, and *p* < 0.05 was considered statistically significant.

## Conflict of Interest

The authors declare no conflict of interest.

## Author Contributions

S.Z., H.S., and J.Y. designed the study. Y.H. performed the experiments. S.Z. and H.S. supervised the study. Y.P., L.Y., C.G., X.W., and Y.H. analyzed and interpreted the data. Y.L. and C.C. performed the statistical analysis. Y.H., S.Z., H.S., and J.Y. wrote the manuscript. All authors read and approved the final manuscript.
